# Complete Chloroplast Genomes of *Papaver rhoeas* and *Papaver orientale*: Molecular Structures, Comparative Analysis, and Phylogenetic Analysis

**DOI:** 10.3390/molecules23020437

**Published:** 2018-02-16

**Authors:** Jianguo Zhou, Yingxian Cui, Xinlian Chen, Ying Li, Zhichao Xu, Baozhong Duan, Yonghua Li, Jingyuan Song, Hui Yao

**Affiliations:** 1 Key Lab of Chinese Medicine Resources Conservation, State Administration of Traditional Chinese Medicine of the People’s Republic of China, Institute of Medicinal Plant Development, Chinese Academy of Medical Sciences & Peking Union Medical College, Beijing 100193, China; jgzhou1316@163.com (J.Z.); yxcui2017@163.com (Y.C.); chenxinlian1053@163.com (X.C.); liying@implad.ac.cn (Y.L.); xuzhichao830@126.com (Z.X.); jysong@implad.ac.cn (J.S.); 2 College of Pharmaceutical Science, Dali University, Dali 671000, China; bzduan@126.com; 3 Department of Pharmacy, Guangxi Traditional Chinese Medicine University, Nanning 530200, China; liyonghua185@126.com

**Keywords:** *Papaver rhoeas*, *Papaver orientale*, chloroplast genome, molecular structure, comparative analysis, phylogenetic analysis

## Abstract

*Papaver rhoeas* L. and *P. orientale* L., which belong to the family Papaveraceae, are used as ornamental and medicinal plants. The chloroplast genome has been used for molecular markers, evolutionary biology, and barcoding identification. In this study, the complete chloroplast genome sequences of *P. rhoeas* and *P. orientale* are reported. Results show that the complete chloroplast genomes of *P. rhoeas* and *P. orientale* have typical quadripartite structures, which are comprised of circular 152,905 and 152,799-bp-long molecules, respectively. A total of 130 genes were identified in each genome, including 85 protein-coding genes, 37 tRNA genes, and 8 rRNA genes. Sequence divergence analysis of four species from Papaveraceae indicated that the most divergent regions are found in the non-coding spacers with minimal differences among three *Papaver* species. These differences include the *ycf1* gene and intergenic regions, such as *rpoB-trnC*, *trnD-trnT*, *petA-psbJ*, *psbE-petL*, and *ccsA-ndhD*. These regions are hypervariable regions, which can be used as specific DNA barcodes. This finding suggested that the chloroplast genome could be used as a powerful tool to resolve the phylogenetic positions and relationships of Papaveraceae. These results offer valuable information for future research in the identification of *Papaver* species and will benefit further investigations of these species.

## 1. Introduction

*Papaver rhoeas* L. and *P. orientale* L. are annual and perennial herbs, respectively, that belong to the family of Papaveraceae [1]. *P. orientale* was first brought to Europe by Tournefort in the early eighteenth century and was introduced as “oriental poppy” [2]. These two species are used as ornamental plants due to their beautiful and showy cup-shaped flowers in various colors and bicolored and semidouble forms [1,3,4]. Chemical studies have shown that these two species contain various alkaloids, including oripavine and thebaine [5,6]. Moreover, these two species are used as treatments for coughs, gastric ulcers, and minor sleep disorders [7,8], thus making them important medicinal plants [9]. Additionally, the seeds, pedicles, and red petals of *P. rhoeas* can be used as food, with the pedicles being commonly used for salads and the red petals for the production of poppy sorbet in Turkey [10]. However, *P. rhoeas* has been shown to cause intoxication in several cases, including central nervous system depression, epileptic seizures, and acute liver toxicity [11,12]. The plants from the genus *Papaver* are similar in their flower-shapes, colors, and fruits, thereby complicating identification based only on morphological characteristics [4]. Previous studies have identified *Papaver* species using physicochemical methods, including amplified fragment length polymorphism [13], discrete stationary wavelet transform–Fourier transform infrared spectroscopy–Radial basis function neural network [14], as well as ice cold water pretreatment and 𝛼-bromonaphthalene cytogenetic methods [4]. Hosokawa et al. [3] authenticated *Papaver* species based on the plastid gene *rpl16* and *rpl16-rpl14* spacer sequences. Liu et al. [15] screened five potential sequences (ITS, *matK*, *psbA-trnH*, *rbcL*, and *trnL-trnF*) to determine candidate sequences that can be used as DNA barcodes to identify the *Papaver* genus, suggesting afterward that *trnL-trnF* can be considered a novel DNA barcode in this genus. The other four sequences can be used as combined barcodes for identification.

Chloroplasts are distinctly important organelles, which have their own genomes. They sustain plant growth and development by converting solar energy to carbohydrates through photosynthesis [16,17,18]. Chloroplast genomes contain valuable information and have been used as ideal research models, particularly for molecular markers, barcoding identification, plant phylogenetics, evolution, and comparative genomic studies [19,20,21]. The highly conserved structure of the chloroplast genome is a potential source of information for the phylogenetic reconstruction of species relationships among plants [22]. A typical circular chloroplast genome has a conserved quadripartite structure consisting of a large single-copy region (LSC) and a small single-copy region (SSC), which are separated by a pair of inverted repeats (IRs). Moreover, the majority of chloroplast genomes of angiosperms are in the range of 120–160 kb in length [23]. The chloroplast genome can be divided into two comprehensive categories, which are namely protein-coding genes and non-coding regions. The latter is further divided into introns and intergenic regions [24]. The first reports examining the complete chloroplast genome sequences from tobacco (*Nicotiana tabacum*) and liverwort (*Marchantia polymorpha*) were reported in 1986 [25,26]. Since then, with the rapid development of next-generation sequencing technology, sequencing the complete chloroplast genome has become inexpensive and efficient compared with the Sanger method [27]. More than 1800 chloroplast genome sequences have been recorded so far in the National Center for Biotechnology Information (NCBI) [28].

A total of 40 genera and approximately 800 species are classified within Papaveraceae, and these are located mainly in the Northern Hemisphere. Of these plants, 19 genera (one endemic and two introduced) and 443 species (295 endemic, five introduced, and one requiring verification) are distributed in China [1]. However, only two species’ chloroplast genome sequences from this family, *Coreanomecon hylomeconoides* [29] and *Papaver somniferum* [30], have been reported. This has hindered our understanding and progress in the research of species identification and phylogeny of Papaveraceae. In this study, we determined the complete chloroplast genome sequences of *P. rhoeas* and *P. orientale*. Furthermore, to discover highly divergent regions of the chloroplast genomes among species from the genus *Papaver*, we compared these two species with *P. somniferum*. The results will provide genetic information on the chloroplast of *P. rhoeas* and *P. orientale* as well as basic knowledge for identifying *Papaver* species.

## 2. Results and Discussion

### 2.1. Features of the Chloroplast Genomes of *P. rhoeas* and *P. orientale*

**Figure 1:**
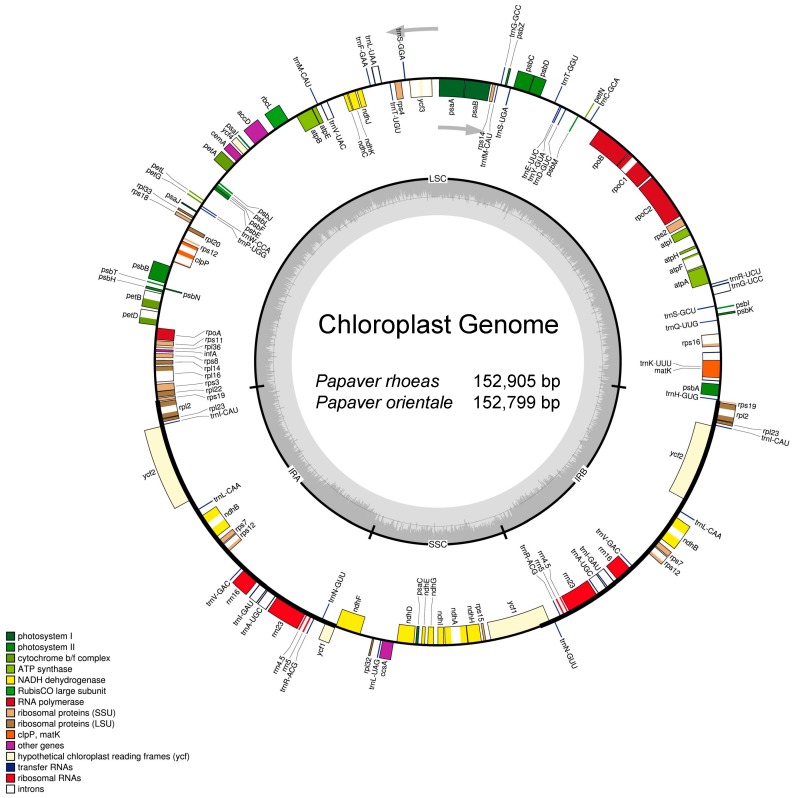
Gene map of the complete chloroplast genomes of *P. rhoeas* and *P. orientale*. Genes inside the circle are transcribed clockwise, whereas those on the outside are transcribed counter-clockwise. The darker gray area in the inner circle corresponds to GC content, whereas the lighter gray area corresponds to AT content.

The complete chloroplast genome sequence of *P. rhoeas* obtained in this research exhibits a typical circular form and encodes 152,905 nucleotides. These nucleotides are encompassed in the quadripartite structure built in four regions (LSC, SSC, IRa, and IRb). The respective four regions occupy 83,172 bp for LSC, 17,971 bp for SSC, and 51,762 bp (25,881 bp each) for the pair of IRs. The gene content, order, and orientation of the chloroplast genome of *P. orientale* are similar to those of *P. rhoeas*. The complete chloroplast genome sequence of *P. orientale* is a circular molecule with a length of 152,799 bp, which is comprised of an LSC region of 83,151 bp and an SSC region of 17,934 bp. These regions are separated by a pair of IRs, each of which have a length of 25,857 bp (Figure 1 and Table 1). The analysis revealed that the average GC contents in the chloroplast genomes of *P. rhoeas* and *P. orientale* are 38.8% and 38.6%, respectively (Table 1). In both species, the IR regions exhibited the highest values of GC content across the complete chloroplast genome (43.2% and 43.1% for *P. rhoeas* and *P. orientale*, respectively). Furthermore, the LSC regions have GC contents of 37.3% and 37.2%, while the lowest values of 33.4% and 33.1% are seen in SSC regions.

A total of 113 functional genes, including 79 protein-coding genes, 30 tRNAs, four rRNAs, and one pseudogene (*ycf1*), were identified from each genome (Table 2). In addition, 17 functional genes are duplicated in the IR regions with a total of 131 genes present in each chloroplast genome. A total of nine genes (*petB*, *petD*, *atpF*, *ndhB*, *ndhA*, *rpoC1*, *rps16*, *rpl16*, and *rpl2*) and six tRNA genes contain one intron, while three genes (*rps12*, *ycf3*, and *clpP*) contain two introns (Table 2). Approximately 51.2% of the complete chloroplast genomes contain protein-coding genes (78,285 bp in *P. rhoeas* and 79,117 bp in *P. orientale*), 5.9% contain rRNAs (9028 bp in both species), and 1.8% contain tRNAs (2788 bp in both species). In contrast, the non-coding regions, including introns, pseudogenes, and intergenic spacers, form 41.1% of the genomes. The basic information and gene contents of the chloroplast genomes of *P. rhoeas* and *P. orientale* compared to four other species, *P. somniferum*, *C. hylomeconoides*, *Arabidopsis thaliana*, and *Nicotiana tabacum*, are presented in Table S1.

### 2.2. Codon Usage Analysis

**Figure 2:**
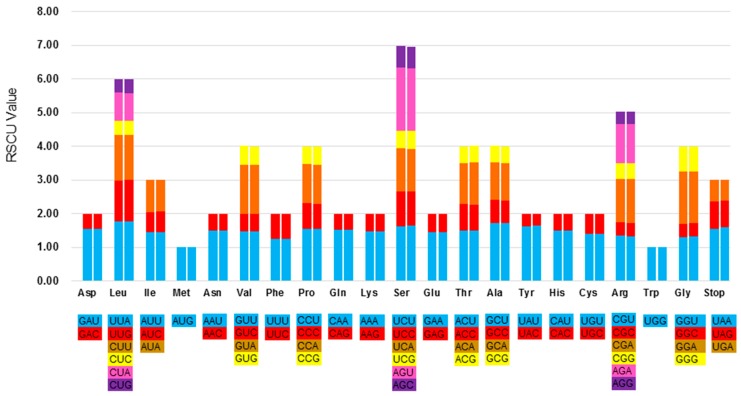
Codon content of 20 amino acid and stop codons in all protein-coding genes of the chloroplast genomes of two *Papaver* species. The histogram on the left hand side of each amino acid shows codon usage within the *P. rhoeas* chloroplast genome, whereas the right hand side illustrates the genome of *P. orientale*.

Relative synonymous codon usage (RSCU) is a measure of non-uniform synonymous codon usage in coding sequences. This is the ratio between frequency of use and expected frequency of a particular codon. RSCU values <1.00 indicate use of a codon less frequent than expected, while codons used more frequently than expected have a score of >1.00 [31]. Based on the sequences of protein-coding genes (CDS), the codon usage frequency was estimated for the chloroplast genomes of *P. rhoeas* and *P. orientale* (summarized in Figure 2 and Tables S2 and S3). The results reveal the presence of 63 codons, which encode 20 amino acids within the chloroplast protein-coding genes of these two species. All the protein-coding genes were composed of 26,039 and 26,095 codons in the chloroplast genomes of *P. rhoeas* and *P. orientale*, respectively. Leucine and cysteine are the most and least abundant universal amino acids in the chloroplast genome of two species, respectively. Other than methionine, amino acid codons in the chloroplast genomes of two species preferentially end with A or U (RSCU > 1). Codons ending in A and/or U accounted for 69.7% and 68.9% of all CDS codons of the chloroplast genomes of *P. rhoeas* and *P. orientale*, respectively. This codon usage pattern is similar to those reported for other chloroplast genomes, which may be driven by a composition bias for a high proportion of A/T. The majority of protein-coding genes in land plant chloroplast genomes employ standard ATG initiator codons. The use of the start codon (ATG) and TGG, encoding Trp, exhibited no bias (RSCU = 1) in these two chloroplast genomes. The findings also revealed that most of the amino acid codons have preferences, with the exception of methionine and tryptophan. Moreover, usage is generally biased toward A or T (U) with high RSCU values, including UUA (1.77) in leucine, UAU (1.63) in tyrosine, and the stop-codon UAA (1.55) in the chloroplast genome of *P. rhoeas* (Table S1). The data presented in Figure 2 illustrated that the RSCU value increases with an increase in the number of codons that code for a specific amino acid. High-codon preference, especially a strong AT bias in codon usage, is very common in other land plant chloroplast genomes [32,33]. The present results were similar to the chloroplast genomes of *Taxillus* [34], *Aristolochia* [21], and *Ulmus* [35] species in terms of codon usage. 

### 2.3. Simple Sequence Repeats and Repeat Structure Analysis

**Figure 3:**
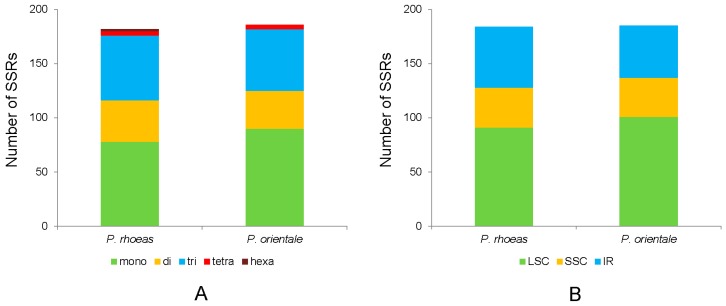
Distribution of simple sequence repeats (SSRs) in the chloroplast genomes of two *Papaver* species. (**A**) SSR type distribution in the chloroplast genomes of two species. (**B**) Proportion of SSRs in different genomic regions of the chloroplast genomes of two species.

Simple sequence repeats (SSRs) are known as microsatellites throughout genomes and comprise tandem repeated DNA sequences that consist of 1–6 repeat nucleotide units [36]. Due to their high levels of polymorphism, SSRs are widely used as molecular markers in species identification, phylogenetic investigations, and population genetics [36,37,38]. A total of 182 and 186 SSRs were detected in the chloroplast genomes of *P. rhoeas* and *P. orientale*, respectively (Table 3; Tables S4 and S5). Mononucleotide repeats were most abundant, which were encountered 78 and 90 times in each case. In comparison, A/T mononucleotide repeats (92.3% and 92.2%, respectively; Table 3) were the most common. No pentanucleotide SSRs existed in these two species. Interestingly, the number of trinucleotide SSRs (60 and 57, respectively) exceeded those of dinucleotide SSRs (38 and 35, respectively). SSRs were more abundant in LSC regions than in IR and SSC regions (Figure 3 and Table 3). Furthermore, almost all SSR loci were composed of A or T, which contributed to the bias in base composition (A/T; 61.2% and 61.4%, respectively) in the chloroplast genomes of two species. 

**Figure 4:**
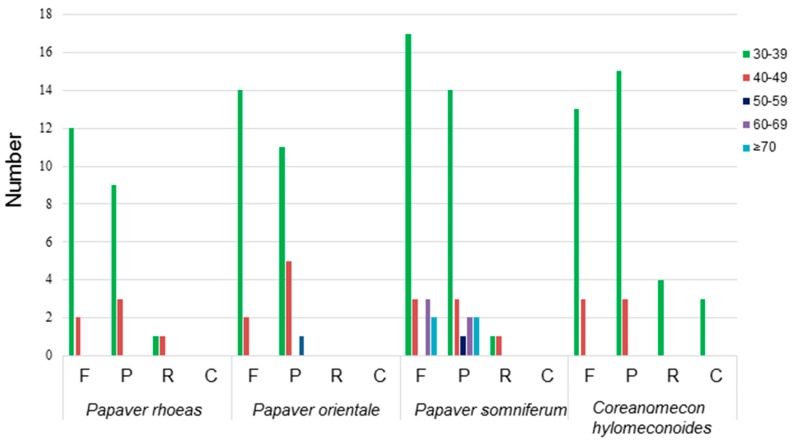
Repeat sequences in four chloroplast genomes. REPuter was used to identify repeat sequences with length ≥30 bp and sequence identified ≥90% in the chloroplast genomes. F, P, R, and C indicate the repeat types F (forward), P (palindrome), R (reverse), and C (complement), respectively. Repeats with different lengths are indicated in different colors.

Dispersed repeat sequences, which play an important role in genome rearrangement, have been used as a source for understanding the phylogenetic relationships of species [39]. They may facilitate intermolecular recombination and create diversity among the chloroplast genomes in a population. These repeats were mostly distributed in the intergenic spacer (IGS) and intron sequences. Repeat sequences with a repeat unit longer than 30 bp were analyzed. Figure 4 shows the repeat structure analyses of four species including three *Papaver* species and *C. hylomeconoides*. The results revealed that the repeats of chloroplast genome of *P. somniferum* had the greatest number, comprising 25 forward, 22 palindromic, and 2 reverse repeats. The second is *C. hylomeconoides*, which contained 16 forward, 18 palindromic, 4 reverse, and 3 complement repeats. The majority of these repeats were mainly forward and palindromic types with lengths mainly in the range of 30–50 bp. The repeats identified in this study will provide valuable information to support investigation of the phylogeny of population studies of these four species.

### 2.4. IR Contraction and Expansion

**Figure 5:**
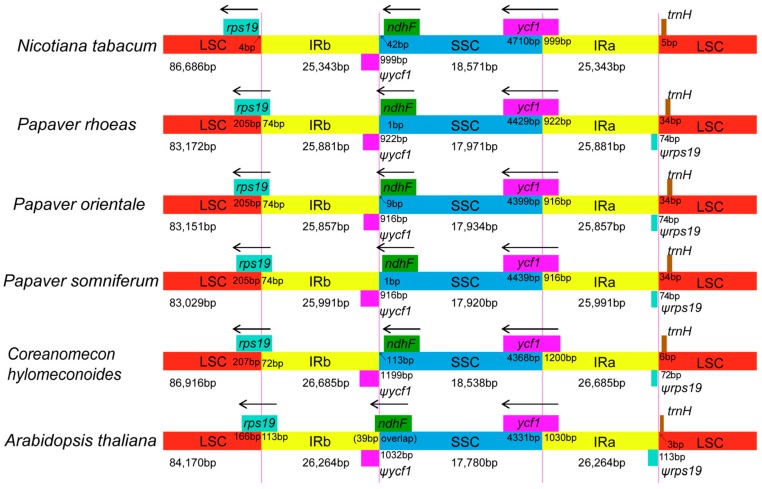
The borders of LSC, SSC and IR regions among six chloroplast genomes. The number above the gene features means the distance between the ends of genes and the borders sites. The IRb/SSC border extended into the *ycf1* genes to create various lengths of *ycf1* pseudogenes among the six chloroplast genomes. The IRb/LSC border extended into the *rps19* genes to create various lengths of *rps19* pseudogenes among the five chloroplast genomes. The arrows indicated the orientation (5’ → 3’) of the *ycf1*, *rps19*, and *ndhF* genes. These features are not to scale.

Genomic structure, including gene number and gene order, is highly conserved among the *Papaver* species. However, structural variation was still present in the LSC/IR/SSC boundaries (Figure 5). We selected two phylogenetically close species (*P. somniferum* and *C. hylomeconoides*) and the model species (*Nicotiana tabacum* and *Arabidopsis thaliana*) as references to compare the chloroplast genome structure. For *P. rhoeas*, the IRa/SSC border was in the 3′ region of the complete *ycf1* gene and created a *ycf1* pseudogene in IRb with a length of 922 bp. The same was found with the *rps19* gene. The LSC/IRb border (position 83,172) was located within the coding region of *rps19*. Correspondingly, a 3′-truncated *rps19* pseudogene with a length of 74 bp was located in the IRa/LSC border (position 152,905). The IRb/LSC border of two other *Papaver* species, *C. hylomeconoides* and *A. thaliana*, were also located within the *rps19* gene. As a result, the *rps19* genes of these species have apparently lost their protein-coding ability because they were partially duplicated in the IRb region and thus produced a pseudogenized *rps19* gene. Only the IRb/SSC border of *A. thaliana* was located in the coding region of the *ndhF* gene.

### 2.5. Comparative Genome Analysis

**Figure 6:**
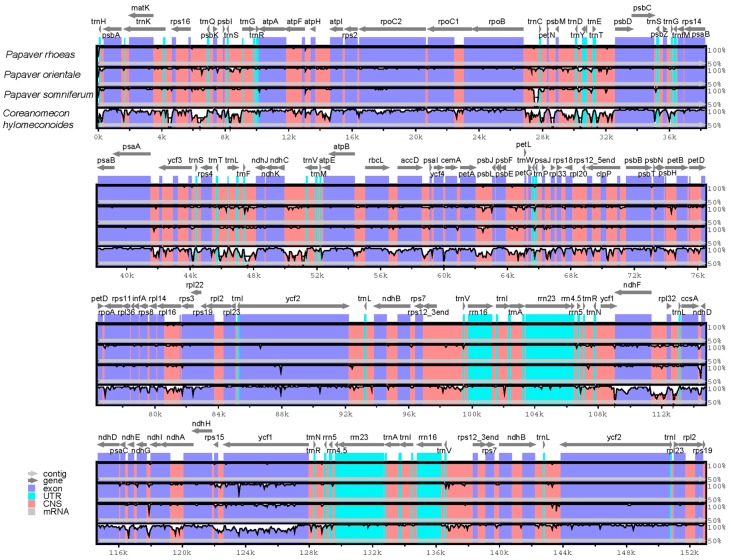
Sequence identity plot comparing the four chloroplast genomes with *P. rhoeas* as a reference by using mVISTA. Gray arrows and thick black lines above the alignment indicate genes with their orientation and the position of the inverted repeats (IRs), respectively. A cut-off of 70% identity was used for the plots, and the Y-scale represents the percent identity ranging from 50 to 100%.

The whole chloroplast genome sequences of *P. rhoeas* and *P. orientale* were compared with those of *P. somniferum* (NC_029434) and *C. hylomeconoides* (NC_031446) using the mVISTA program (Figure 6). The comparison showed few differences among the chloroplast genomes of the three *Papaver* species. These differences included the *ycf1* gene and intergenic regions, such as *rpoB-trnC*, *trnD-trnT*, *petA-psbJ*, *psbE-petL*, and *ccsA-ndhD*. These regions are hypervariable regions, which can be used as specific DNA barcodes. Additionally, two IR regions were less divergent than the LSC and SSC regions. The four rRNA genes were the most conserved and had almost no difference among the three *Papaver* species. Additionally, the results revealed that non-coding regions exhibit a higher divergence than coding regions, with the most divergent regions localized in the IGSs among the four chloroplast genomes.

**Figure 7:**
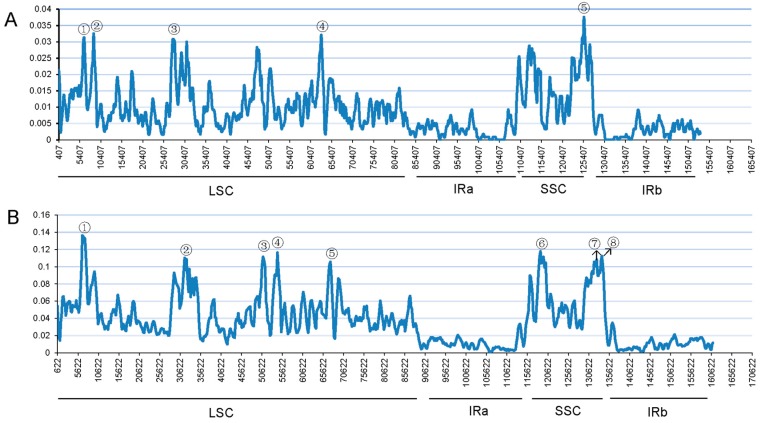
Sliding window analysis of the whole chloroplast genomes. Window length: 800 bp; step size: 200 bp. X-axis: position of the midpoint of a window. Y-axis: nucleotide diversity of each window. (**A**) Pi among three *Papaver* species. (**B**) Pi among three *Papaver* species and *C. hylomeconoides*. ①–⑧ indicate mutational hotspots and highly divergent loci.

Furthermore, sliding window analysis using DnaSP detected highly variable regions in the chloroplast genomes of three *Papaver* species and *C. hylomeconoides*. The nucleotide variability (Pi) was calculated to show divergence at the sequence level (Figure 7). Figure 7A shows that the average value of Pi was 0.00895 among the three *Papaver* species. As expected, the IR regions exhibited lower variability than the LSC and SSC regions. Five mutational hotspots were observed, which showed remarkably higher Pi values (>0.03) and were located at the LSC and SSC regions. Figure 7B shows that the average value of Pi was 0.03761 among the four species, including three *Papaver* species and *C. hylomeconoides*. The Pi values of these four species were commonly higher than those of the three *Papaver* species. Particularly, eight highly divergent loci showed remarkably higher Pi values (>0.1). These regions may be undergoing rapid nucleotide substitution at the species level, indicating potential application of molecular markers for plant identification and phylogenetic analysis.

### 2.7. Phylogenetic Analysis

**Figure 8:**
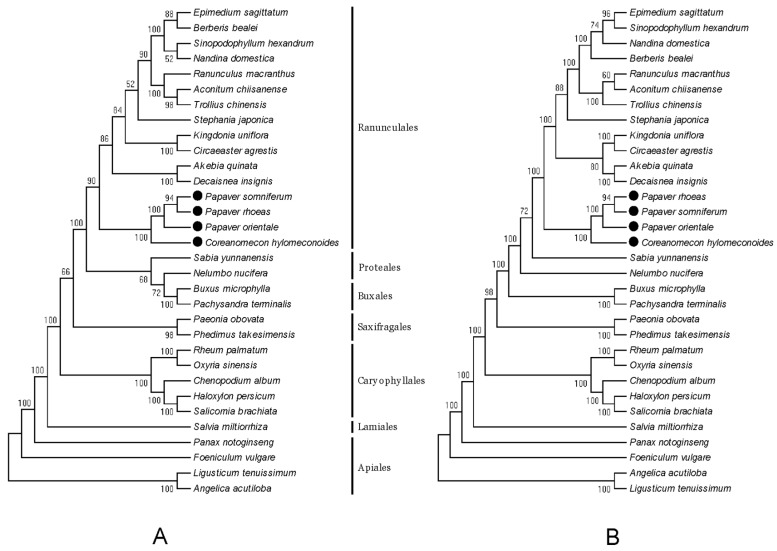
Phylogenetic trees constructed with 54 protein-coding genes of 32 species using maximum likelihood (ML) and maximum parsimony (MP) methods. Numbers at nodes are values for bootstrap support. These trees are unrooted cladograms. (**A**) ML tree; (**B**) MP tree.

Recent advances in high-throughput sequencing have provided large amounts of data, improving phylogenetic resolution. The chloroplast genome has been widely employed as an important source of molecular markers in plant systematics. In this study, to determine the phylogenetic position of *P. rhoeas* and *P. orientale*, 30 complete chloroplast genome sequences were obtained from GenBank. The maximum likelihood (ML) and maximum parsimony (MP) trees exhibited similar phylogenetic topologies (Figure 8). The results illustrated that two *Papaver* species were the closest sister species of *P. somniferum*. These three species were grouped with *C. hylomeconoides*. These four species from the family of Papaveraceae were sister taxa with respect to two species from Lardizabalaceae (*Akebia quinata* and *Decaisnea insignis*) and two species from Circaeasteraceae (*Kingdonia uniflora* and *Circaeaster agrestis*) within Ranunculales. Both ML and MP trees showed that species from Ranunculales were grouped with Proteales. This result (inferred from the chloroplast genome data) obtained high support values, which suggested that the chloroplast genome could be used as a powerful tool to resolve the phylogenetic positions and relationships of Papaveraceae. Nevertheless, to accurately illustrate the evolution of the family Papaveraceae, using more species to analyze the phylogeny is necessary. This study will also provide a reference for species identification among *Papaver* and other genera using the chloroplast genome.

## 3. Materials and Methods

### 3.1. Plant Material, DNA Extraction, and Sequencing

Fresh plants of *P. rhoeas* and *P. orientale* were collected from the Beijing Medicinal Plant Garden. All samples were identified by Professor Yulin Lin, who was based at the Institute of Medicinal Plant Development (IMPLAD), the Chinese Academy of Medical Sciences (CAMS), and the Peking Union Medical College (PUMC). The voucher specimens were deposited in the herbarium of IMPLAD. Total genomic DNA was extracted from the clean leaves of samples frozen at −80 °C using DNeasy Plant Mini Kit with a standard protocol (Qiagen Co., Hilden, Germany), and DNA quality was assessed based on spectrophotometry and electrophoresis in 1% (w/v) agarose gel. The DNA was used to generate shotgun libraries with an average insert size of 500 bp and sequenced using the Illumina Hiseq X (v2, Illumina, San Diego CA, USA) in accordance with the standard protocol. Approximately 6.3 GB of raw data from *P. rhoeas* and 6.6 GB from *P. orientale* were generated with 150 bp paired-end read lengths.

### 3.2. Chloroplast Genome Assembly and Annotation

First, the low-quality reads were trimmed from the raw reads using Trimmomatic V0.36 [40]. After this, the clean reads were mapped to the database, which was constructed from all chloroplast genome sequences recorded in the NCBI on the basis of their coverage and similarity. Finally, the mapped reads were assembled to contigs using SOAPdenovo2 [41]. SSPACE [42] was used to construct the scaffold of the chloroplast genome, and GapCloser was used to fill the gaps [41]. To verify the assembly, four boundaries between single copy (SC) and inverted repeat (IR) regions of the assembled sequences were confirmed by PCR amplification and Sanger sequencing using the primers listed in Table S6.

Annotation of the complete chloroplast genomes was executed using the online program Dual Organellar GenoMe Annotator (DOGMA, http://dogma.ccbb.utexas.edu/) [43] and CPGAVAS coupled with manual corrections [44]. The software tRNAscan-SE was used to identify tRNA genes. The circular chloroplast genome map was generated by the Organellar Genome DRAW (OGDRAW) V1.2 [45]. The complete and correct chloroplast genome sequences of the two species were deposited in GenBank. The accession numbers of *P. rhoeas* and *P. orientale* are MF943221 and MF943222, respectively.

### 3.3. Genome Structure Analysis and Genome Comparison

GC content was analyzed using the software MEGA6.0 [46]. The distribution of codon usage was investigated using the software CodonW with the RSCU ratio [31]. The online software MISA [47] was used to detect SSRs with parameters set to be similar to those of Li et al. [48]. REPuter [49] was used to identify the size and location of repeat sequences, including forward, palindromic, reverse, and complement repeats in the chloroplast genomes of four species. For all repeat types, the minimal size was 30 bp and the two repeat copies had at least 90% similarity. Whole-genome alignment for the chloroplast genomes of the four species, three *Papaver* species and *C. hylomeconoides*, was performed and plotted using the mVISTA program [50]. To determine the nucleotide diversity of the chloroplast genome, we analyzed the sliding window using DnaSP v5.10 [51]. The step size was set to 200 bp with an 800 bp window length.

### 3.4. Phylogenetic Analysis

For phylogenetic analysis, 30 complete chloroplast genome sequences were downloaded from the NCBI Organelle Genome Resources database (Table S7). These species are close taxa to Papaveraceae according to traditional classification. The sequences of 54 protein-coding genes commonly presented in 32 species, including the two species in this study, were aligned using the Clustal algorithm [52]. We analyzed these 54 genes to determine the phylogenetic positions of *P. rhoeas* and *P. orientale*. ML analysis was conducted based on the Tamura-Nei model using a heuristic search for initial trees. This model was determined to be the most appropriate by Modeltest [53]. MP analysis was performed with PAUP*4.0b10 [54]. Bootstrap analysis was performed with 1000 replicates.

## 4. Conclusions

The complete chloroplast genome sequences of *P. rhoeas* and *P. orientale* were determined in this study. The results revealed that the size, structure, gene content, and compositional organization are highly conserved among the three *Papaver* species including *P. rhoeas*, *P. orientale*, and *P. somniferum*. Comparison analysis of the three Papaver species and *C. hylomeconoides* revealed genomic diversity, and molecular markers were developed. The results provide a basis for identifying *Papaver* species. The data obtained in this study will open up further avenues of research, based on which more genomic information about the chloroplasts in *Papaver* species can be obtained.

## Figures and Tables

**Table 1:** Base composition in the chloroplast genomes of *P. rhoeas* and *P. orientale*.

Species	Regions	Positions	T(U) (%)	C (%)	A (%)	G (%)	Length (bp)
*P. rhoeas*	LSC		31.9	19.2	30.8	18.1	83,172
	SSC		33.3	17.8	33.3	15.6	17,971
	IRa		28.6	22.2	28.3	21.0	25,881
	IRb		28.3	21.0	28.6	22.2	25,881
	Total		30.9	19.8	30.3	19.0	152,905
	CDS ^1^		31.0	18.0	30.4	20.6	78,285
		1st position ^2^	23.5	18.9	30.4	27.2	26,095
		2nd position ^3^	32.1	20.6	29.2	18.1	26,095
		3rd position ^4^	37.4	14.6	31.5	16.5	26,095
*P. orientale*	LSC		32.0	19.1	30.9	18.1	83,151
	SSC		33.4	17.7	33.5	15.4	17,934
	IRa		28.6	22.2	28.3	20.9	25,857
	IRb		28.3	20.9	28.6	22.2	25,857
	Total		31.0	19.7	30.4	18.9	152,799
	CDS		31.1	18.0	30.4	20.6	78,117
		1st position	23.5	18.9	30.4	27.2	26,039
		2nd position	32.2	20.5	29.2	18.1	26,039
		3rd position	37.5	14.6	31.5	16.4	26,039

^1^ CDS: protein-coding regions; ^2^ 1st position: 1st base of codons; ^3^ 2nd position: 2nd base of codons; ^4^ 3rd position: 3rd base of codons.

**Table 2:** Gene contents in the chloroplast genomes of *P. rhoeas* and *P. orientale*.

Classificaion of Genes	Gene Names	Number of Genes
Photosystem I	*psaA*, *psaB*, *psaC*, *psaI*, *psaJ*	5
Photosystem II	*psbA*, *psbB*, *psbC*, *psbD*, *psbE*, *psbF*, *psbH*, *psbI*, *psbJ*, *psbK*, *psbL*, *psbM*, *psbN*, *psbT*, *psbZ*	15
Cytochrome b/f complex	*petA*, *petB* *, *petD* *, *petG*, *petL*, *petN*	6
ATP synthase	*atpA*, *atpB*, *atpE*, *atpF* *, *atpH*, *atpI*	6
NADH dehydrogenase	*ndhA* *, *ndhB* *(×2), *ndhC*, *ndhD*, *ndhE*, *ndhF*, *ndhG*, *ndhH*, *ndhI*, *ndhJ*, *ndhK*	12
RubisCO large subunit	*rbcL*	1
RNA polymerase	*rpoA*, *rpoB*, *rpoC1* *, *rpoC2*	4
Ribosomal proteins (SSU)	*rps2*, *rps3*, *rps4*, *rps7*(×2), *rps8*, *rps11*, *rps12* **(×2), *rps14*, *rps15*, rps16 *, *rps18*, *rps19*	14
Ribosomal proteins (LSU)	*rpl2* *(×2), *rpl14*, *rpl16* *, *rpl20*, *rpl22*, *rpl23*(×2), *rpl32*, *rpl33*, *rpl36*	11
Ribosomal RNAs	*rrn4.5(×2), rrn5(×2), rrn16(×2), rrn23(×2)*	8
Proteins of unknown function	*ycf1*(×2), *ycf2*(×2), *ycf3* **, *ycf4*	6
Transfer RNAs	37 tRNAs (6 contain an intron, 7 in the inverted repeats (IRs))	37
Other genes	*accD*, *clpP* **, *matK*, *ccsA*, *cemA*, *infA*	6

* Gene contains one intron; ** gene contains two introns; (×2) indicates the number of the repeat unit is 2.

**Table 3:** Types and amounts of SSRs in the chloroplast genomes of *P. rhoeas* and *P. orientale*.

SSR Type	Repeat Unit	Amount		Ratio(%)	
		*P. rhoeas*	*P. orientale*	*P. rhoeas*	*P. orientale*
Mono	A/T	72	83	92.3	92.2
	C/G	6	7	7.7	7.8
Di	AG/CT	20	18	52.6	51.4
	AT/AT	16	15	42.1	42.9
	AC/GT	2	2	5.3	5.7
Tri	AAG/CTT	25	25	41.7	43.9
	AAT/ATT	12	12	20.0	21.1
	AAC/GTT	8	8	13.3	14.0
	ACC/GGT	3	1	5.0	1.7
	ACT/AGT	1	1	1.7	1.7
	AGC/CTG	5	5	8.3	8.8
	AGG/CCT	3	2	5.0	3.5
	ATC/ATG	3	3	5.0	5.3
Tetra	AAAC/GTTT	1	1	25.0	25.0
	AAAT/ATTT	1	1	25.0	25.0
	AACC/GGTT	1	1	25.0	25.0
	AGAT/ATCT	1	1	25.0	25.0
Hexa	AAGAAT/ATTCTT	2	0	100.0	0.0

## Supplementary Materials

The following are available online, Table S1: Comparisons among the chloroplast genome characteristics of *P. rhoeas*, *P. orientale*, *P. somniferum*, *C. hylomeconoides*, *A. thaliana*, and *N. tabacum*, Table S2: Codon usage within the chloroplast genomes of *P. rhoeas*, Table S3: Codon usage within the chloroplast genomes of *P. orientale*, Table S4: Simple sequence repeats (SSRs) in the chloroplast genome of *P. rhoeas*, Table S5: Simple sequence repeats (SSRs) in the chloroplast genome of *P. orientale*, Table S6: Primer sequences at the boundaries between single copy and IR regions, Table S7: GenBank accession numbers of dicots with complete chloroplast genome sequences used for phylogenetic analyses.

## Abbreviations

LSC	large single copy
SSC	small single copy
IR	inverted repeat
SSR	simple sequence repeats
