# Supplementary material for: Complete Chloroplast Genomes of Papaver rhoeas and Papaver orientale: Molecular Structures, Comparative Analysis, and Phylogenetic Analysis
Source: Molecules. 2018 Feb 16;23(2):437. doi: 10.3390/molecules23020437 (PMC6017017; doi:10.3390/molecules23020437)
Supplement: Supplementary file 1 [file molecules-23-00437-s001.pdf]

Article

Complete Chloroplast Genomes of *Papaver rhoeas* and *Papaver orientale*: Molecular Structures, Comparative Analysis, and Phylogenetic Analysis

Jianguo Zhou[1], Yingxian Cui[1], Xinlian Chen[1], Ying Li[1], Zhichao Xu[1], Baozhong Duan[2], Yonghua Li[3], Jingyuan Song[1] and Hui Yao[1,*]

[1] Key Lab of Chinese Medicine Resources Conservation, State Administration of Traditional Chinese Medicine of the People's Republic of China, Institute of Medicinal Plant Development, Chinese Academy of Medical Sciences & Peking Union Medical College, Beijing, 100193, China. jgzhou1316@163.com (J.Z.); yxcui2017@163.com (Y.C.); chenxinlian1053@163.com (X.C.); liying@implad.ac.cn (Y.L.); zcxu@implad.ac.cn (Z.X.); jysong@implad.ac.cn (J.S.)

[2] College of Pharmaceutical Science, Dali University, Dali, 671000, Yunnan, China; bzduan@126.com (B.D.)

[3] Department of Pharmacy, Guangxi Traditional Chinese Medicine University, Nanning 530200, China; liyonghua185@126.com

* Correspondence: scauyaoh@sina.com; Tel.: +86-10-57833194.

Supplementary Materials

Table S1. Comparisons among the chloroplast genome characteristics of *P. rhoeas*, *P. orientale*, *P. somniferum* , *C. hylomeconoides*, *A. thaliana*, and *N. tabacum*.
Table S2. Codon usage within the chloroplast genomes of *P. rhoeas.*
Table S3. Codon usage within the chloroplast genomes of *P. orientale.*
Table S4: Simple sequence repeats (SSRs) in the chloroplast genome of *P. rhoeas*.
Table S5: Simple sequence repeats (SSRs) in the chloroplast genome of *P. orientale*.
Table S6: Primer sequences at the boundaries between single copy and IR regions.
Table S7: GenBank accession numbers of dicots with complete chloroplast genome sequences used for phylogenetic analyses.

Table S1. Comparisons among the chloroplast genome characteristics of *P. somniferum* , *C. hylomeconoides*, *A. thaliana*, and *N. tabacum*.

Species	*P. rhoeas*	*P. orientale*	*P. somniferum*	*C. hylomeconoides*	*A. thaliana*	*N. tabacum*
Family	Papaveraceae	Papaveraceae	Papaveraceae	Papaveraceae	Cruciferae	Solanaceae
Accession No.	MF943221	MF943222	KU204905	KT274030	KX551970	Z00044
Genome size (bp)	152,905	152,799	152,931	158,824	154,515	155,844
LSC length (bp)	83,172	83,151	83,029	86,916	84,170	86,684
SSC length (bp)	17,971	17,934	17,920	18,538	17780	18,482
IR length (bp)	25,881	25,857	25,991	26,685	26264	25,339
GC content (%)	38.8	38.6	38.9	38.8	36.3	37.8
Number of genes	130	130	130	130	132	146
Number of protein-coding genes	85	85	85	85	87	101
Number of tRNAs	37	37	37	37	37	37
Number of rRNAs	8	8	8	8	8	8

Table S2. Codon usage within the chloroplast genomes of *P. rhoeas*.

Amino acid	Codon	No.	RSCU	tRNA	Amino acid	Codon	No.	RSCU	tRNA
Phe	UUU	906	1.26		Tyr	UAU	775	1.63	
Phe	UUC	537	0.74	*trnF-GAA*	Tyr	UAC	174	0.37	*trnY-GUA*
Leu	UUA	797	1.77	*trnL-UAA*	Stop	UAA	44	1.55	
Leu	UUG	551	1.22	*trnL-CAA*	Stop	UAG	23	0.81	
Leu	CUU	602	1.34		His	CAU	480	1.51	
Leu	CUC	196	0.43		His	CAC	154	0.49	*trnH-GUG*
Leu	CUA	375	0.83	*trnL-UAG*	Gln	CAA	686	1.52	*trnQ-UUG*
Leu	CUG	183	0.41		Gln	CAG	218	0.48	
Ile	AUU	1038	1.45		Asn	AAU	936	1.51	
Ile	AUC	434	0.6	*trnI-GAU*	Asn	AAC	306	0.49	*trnN-GUU*
Ile	AUA	683	0.95	*trnI-CAU*	Lys	AAA	1016	1.48	*trnK-UUU*
Met	AUG	624	1	*trn(f)M-CAU*	Lys	AAG	356	0.52	
Val	GUU	537	1.48		Asp	GAU	849	1.55	
Val	GUC	185	0.51	*trnV-GAC*	Asp	GAC	246	0.45	*trnD-GUC*
Val	GUA	533	1.47	*trnV-UAC*	Glu	GAA	971	1.44	*trnE-UUC*
Val	GUG	199	0.55		Glu	GAG	373	0.56	
Ser	UCU	559	1.63		Cys	UGU	219	1.41	
Ser	UCC	349	1.02	*trnS-GGA*	Cys	UGC	91	0.59	*trnC-GCA*
Ser	UCA	444	1.3	*trnS-UGA*	Stop	UGA	18	0.64	
Ser	UCG	174	0.51		Trp	UGG	468	1	*trnW-CCA*
Pro	CCU	424	1.55		Arg	CGU	357	1.34	*trnR-ACG*
Pro	CCC	210	0.77		Arg	CGC	105	0.4	
Pro	CCA	318	1.16	*trnP-UGG*	Arg	CGA	342	1.29	
Pro	CCG	141	0.52		Arg	CGG	122	0.46	
Thr	ACU	500	1.5		Arg	AGA	397	1.16	*trnR-UCU*
Thr	ACC	262	0.78	*trnT-GGU*	Arg	AGG	132	0.39	
Thr	ACA	408	1.22	*trnT-UGU*	Ser	AGU	497	1.87	
Thr	ACG	167	0.5		Ser	AGC	170	0.64	*trnS-GCU*
Ala	GCU	597	1.73		Gly	GGU	588	1.31	
Ala	GCC	235	0.68		Gly	GGC	175	0.39	*trnG-GCC*
Ala	GCA	386	1.12	*trnA-UGC*	Gly	GGA	690	1.54	*trnG-UCC*
Ala	GCG	166	0.48		Gly	GGG	341	0.76	

Table S3. Codon usage within the chloroplast genomes of *P. orientale*.

Amino acid	Codon	No.	RSCU	tRNA	Amino acid	Codon	No.	RSCU	tRNA
Phe	UUU	901	1.25		Tyr	UAU	770	1.64	
Phe	UUC	538	0.75	*trnF-GAA*	Tyr	UAC	170	0.36	*trnY-GUA*
Leu	UUA	799	1.77	*trnL-UAA*	Stop	UAA	45	1.59	
Leu	UUG	554	1.23	*trnL-CAA*	Stop	UAG	23	0.81	
Leu	CUU	599	1.33		His	CAU	485	1.51	
Leu	CUC	200	0.44		His	CAC	156	0.49	*trnH-GUG*
Leu	CUA	366	0.81	*trnL-UAG*	Gln	CAA	688	1.52	*trnQ-UUG*
Leu	CUG	189	0.42		Gln	CAG	218	0.48	
Ile	AUU	1040	1.45		Asn	AAU	927	1.49	
Ile	AUC	441	0.61	*trnI-GAU*	Asn	AAC	314	0.51	*trnN-GUU*
Ile	AUA	678	0.94	*trnI-CAU*	Lys	AAA	1010	1.47	*trnK-UUU*
Met	AUG	624	1	*trn(f)M-CAU*	Lys	AAG	367	0.53	
Val	GUU	540	1.48		Asp	GAU	849	1.55	
Val	GUC	186	0.51	*trnV-GAC*	Asp	GAC	250	0.45	*trnD-GUC*
Val	GUA	530	1.45	*trnV-UAC*	Glu	GAA	973	1.44	*trnE-UUC*
Val	GUG	204	0.56		Glu	GAG	378	0.56	
Ser	UCU	562	1.64		Cys	UGU	215	1.39	
Ser	UCC	345	1.01	*trnS-GGA*	Cys	UGC	95	0.61	*trnC-GCA*
Ser	UCA	439	1.28	*trnS-UGA*	Stop	UGA	17	0.6	
Ser	UCG	181	0.53		Trp	UGG	468	1	*trnW-CCA*
Pro	CCU	424	1.54		Arg	CGU	354	1.33	*trnR-ACG*
Pro	CCC	207	0.75		Arg	CGC	103	0.39	
Pro	CCA	323	1.17	*trnP-UGG*	Arg	CGA	348	1.31	
Pro	CCG	147	0.53		Arg	CGG	122	0.46	
Thr	ACU	502	1.5		Arg	AGA	398	1.16	*trnR-UCU*
Thr	ACC	257	0.77	*trnT-GGU*	Arg	AGG	133	0.39	
Thr	ACA	418	1.25	*trnT-UGU*	Ser	AGU	495	1.86	
Thr	ACG	163	0.49		Ser	AGC	171	0.64	*trnS-GCU*
Ala	GCU	603	1.72		Gly	GGU	591	1.32	
Ala	GCC	234	0.67		Gly	GGC	176	0.39	*trnG-GCC*
Ala	GCA	393	1.12	*trnA-UGC*	Gly	GGA	692	1.54	*trnG-UCC*
Ala	GCG	169	0.48		Gly	GGG	338	0.75	

Table S4. Simple sequence repeats (SSRs) in the chloroplast genome of *P. rhoeas*.

SSR type	SSR	size(bp)	start	end	Region
p3	(AAG)3	9	599	607	LSC
p1	(A)8	8	1606	1613	LSC
p3	(GAA)3	9	2014	2022	LSC
p1	(T)9	9	2188	2196	LSC
p3	(GAA)3	9	2826	2834	LSC
p1	(T)8	8	3210	3217	LSC
p3	(ATA)3	9	3550	3558	LSC
p1	(A)8	8	6087	6094	LSC
p1	(A)12	12	7182	7193	LSC
p1	(T)9	9	8019	8027	LSC
p1	(A)8	8	8093	8100	LSC
p3	(AAC)3	9	8510	8518	LSC
p1	(T)8	8	9325	9332	LSC
p1	(T)9	9	9677	9685	LSC
p1	(A)8	8	13216	13223	LSC
p4	(AAAT)3	12	13682	13693	LSC
p4	(GGTT)3	12	14271	14282	LSC
p3	(AAC)3	9	14773	14781	LSC
p3	(TTA)3	9	15582	15590	LSC
p1	(T)9	9	18410	18418	LSC
p1	(T)11	11	18516	18526	LSC
p1	(A)8	8	18659	18666	LSC
p2	(AT)4	8	19799	19806	LSC
p2	(AT)5	10	19889	19898	LSC
p3	(ATT)3	9	21148	21156	LSC
p3	(TTC)3	9	22143	22151	LSC
p1	(A)8	8	22327	22334	LSC
p1	(T)8	8	26022	26029	LSC
p1	(T)8	8	26128	26135	LSC
p3	(TAT)3	9	27041	27049	LSC
p2	(AT)5	10	27370	27379	LSC
p1	(A)8	8	27695	27702	LSC
p1	(A)8	8	29610	29617	LSC
p4	(AGAT)3	12	29678	29689	LSC
p2	(CT)4	8	30066	30073	LSC
p3	(TTA)3	9	30225	30233	LSC
p1	(A)8	8	31063	31070	LSC
p3	(TTA)3	9	31430	31438	LSC
p2	(GA)5	10	32330	32339	LSC
p1	(G)8	8	34191	34198	LSC
p2	(GA)4	8	35229	35236	LSC
p3	(TCT)3	9	36840	36848	LSC

p3	(TAT)3	9	36891	36899	LSC
p3	(ATG)3	9	38383	38391	LSC
p3	(TTG)3	9	39988	39996	LSC
p3	(GCA)3	9	40281	40289	LSC
p3	(ACC)3	9	41099	41107	LSC
p1	(T)8	8	41759	41766	LSC
p4	(AACA)3	12	44487	44498	LSC
p2	(TA)4	8	45481	45488	LSC
p2	(AT)4	8	46141	46148	LSC
p1	(T)8	8	46767	46774	LSC
p2	(AG)4	8	47573	47580	LSC
p2	(TA)4	8	47816	47823	LSC
p1	(T)8	8	47910	47917	LSC
p1	(A)8	8	50156	50163	LSC
p1	(T)8	8	51463	51470	LSC
p1	(T)12	12	54640	54651	LSC
p3	(AGA)3	9	57777	57785	LSC
p2	(TC)4	8	60180	60187	LSC
p1	(T)8	8	60887	60894	LSC
p1	(A)9	9	61465	61473	LSC
p2	(AC)4	8	62016	62023	LSC
p1	(A)11	11	62652	62662	LSC
p2	(TC)4	8	63342	63349	LSC
p2	(TA)4	8	64113	64120	LSC
p3	(GTA)3	9	64154	64162	LSC
p1	(T)9	9	65041	65049	LSC
p3	(CCT)3	9	65538	65546	LSC
p2	(GT)4	8	66998	67005	LSC
p3	(AAC)3	9	67299	67307	LSC
p3	(ATT)3	9	67574	67582	LSC
p2	(TA)4	8	67617	67624	LSC
p1	(A)8	8	70279	70286	LSC
p1	(T)8	8	70324	70331	LSC
p1	(T)9	9	71140	71148	LSC
p1	(T)8	8	73914	73921	LSC
p3	(GGA)3	9	75397	75405	LSC
p1	(T)8	8	76877	76884	LSC
p2	(TA)4	8	77064	77071	LSC
p1	(T)8	8	77949	77956	LSC
p1	(A)10	10	78745	78754	LSC
p3	(GGA)3	9	79023	79031	LSC
p1	(T)9	9	79565	79573	LSC
p1	(T)8	8	81354	81361	LSC
p2	(CT)4	8	82478	82485	LSC

p2	(TA)4	8	82504	82511	LSC
p3	(TGC)3	9	82720	82728	LSC
p3	(TTA)4	12	82953	82964	LSC
p1	(T)9	9	83224	83232	IRa
p3	(CTT)3	9	83366	83374	IRa
p1	(T)9	9	84806	84814	IRa
p2	(GA)4	8	85452	85459	IRa
p2	(GA)4	8	85464	85471	IRa
p2	(GA)4	8	86451	86458	IRa
p3	(CAA)3	9	86683	86691	IRa
p1	(G)8	8	87209	87216	IRa
p1	(A)9	9	87352	87360	IRa
p2	(GA)4	8	88603	88610	IRa
p3	(TCT)3	9	89292	89300	IRa
p3	(CTT)3	9	89404	89412	IRa
p3	(GGT)3	9	89668	89676	IRa
p3	(TGA)3	9	89910	89918	IRa
p1	(A)8	8	89958	89965	IRa
p3	(GAA)3	9	91125	91133	IRa
p2	(TA)4	8	91950	91957	IRa
p3	(AGA)3	9	94516	94524	IRa
p3	(AGA)3	9	95975	95983	IRa
p6	(TTCTTA)3	18	96244	96261	IRa
p3	(AAG)3	9	99195	99203	IRa
p1	(C)8	8	99590	99597	IRa
p2	(AG)4	8	101510	101517	IRa
p3	(CTG)3	9	103574	103582	IRa
p2	(CT)4	8	105121	105128	IRa
p1	(A)8	8	106866	106873	IRa
p1	(T)8	8	107263	107270	IRa
p3	(CAA)3	9	108611	108619	IRa
p1	(A)8	8	109095	109102	SSC
p1	(A)8	8	109765	109772	SSC
p1	(T)8	8	109785	109792	SSC
p1	(T)8	8	109872	109879	SSC
p3	(AAT)3	9	110211	110219	SSC
p3	(TAA)3	9	110708	110716	SSC
p1	(T)9	9	111294	111302	SSC
p3	(TTA)4	12	111531	111542	SSC
p1	(A)9	9	112035	112043	SSC
p1	(T)8	8	112759	112766	SSC
p1	(C)8	8	115512	115519	SSC
p1	(A)8	8	115569	115576	SSC
p3	(TTC)3	9	119009	119017	SSC

p1	(T)8	8	119878	119885	SSC
p3	(AGC)3	9	120139	120147	SSC
p2	(TC)5	10	121404	121413	SSC
p1	(T)10	10	121986	121995	SSC
p2	(AT)4	8	122620	122627	SSC
p1	(T)13	13	123027	123039	SSC
p1	(T)10	10	123558	123567	SSC
p2	(TA)4	8	123650	123657	SSC
p1	(A)8	8	123747	123754	SSC
p2	(AT)4	8	124015	124022	SSC
p1	(T)11	11	124685	124695	SSC
p3	(CTT)3	9	124706	124714	SSC
p2	(AT)4	8	124822	124829	SSC
p3	(CTT)3	9	124882	124890	SSC
p1	(T)9	9	125057	125065	SSC
p1	(T)11	11	125228	125238	SSC
p1	(T)9	9	125341	125349	SSC
p1	(T)9	9	125351	125359	SSC
p3	(TTC)3	9	125836	125844	SSC
p1	(A)9	9	125963	125971	SSC
p3	(TTC)3	9	126408	126416	SSC
p1	(T)10	10	126562	126571	SSC
p1	(T)13	13	126736	126748	SSC
p1	(A)10	10	126900	126909	SSC
p3	(TTG)3	9	127459	127467	IRb
p1	(A)8	8	128808	128815	IRb
p1	(T)8	8	129205	129212	IRb
p2	(AG)4	8	130950	130957	IRb
p3	(CAG)3	9	132496	132504	IRb
p2	(TC)4	8	134560	134567	IRb
p1	(G)8	8	136481	136488	IRb
p3	(CTT)3	9	136875	136883	IRb
p6	(TAAGAA)3	18	139817	139834	IRb
p3	(TTC)3	9	140094	140102	IRb
p3	(TCT)3	9	141554	141562	IRb
p2	(TA)4	8	144121	144128	IRb
p3	(TTC)3	9	144945	144953	IRb
p1	(T)8	8	146113	146120	IRb
p3	(TCA)3	9	146160	146168	IRb
p3	(ACC)3	9	146402	146410	IRb
p3	(AAG)3	9	146666	146674	IRb
p3	(AGA)3	9	146778	146786	IRb
p2	(TC)4	8	147468	147475	IRb
p1	(T)9	9	148718	148726	IRb

p1	(C)8	8	148862	148869	IRb
p3	(TTG)3	9	149387	149395	IRb
p2	(CT)4	8	149619	149626	IRb
p2	(TC)4	8	150607	150614	IRb
p2	(TC)4	8	150619	150626	IRb
p1	(A)9	9	151264	151272	IRb
p3	(GAA)3	9	152703	152711	IRb
p1	(A)9	9	152846	152854	IRb

Table S5. Simple sequence repeats (SSRs) in the chloroplast genome of *P. orientale*.

SSR type	SSR	size(bp)	start	end	Region
p1	(A)10	10	1600	1609	LSC
p3	(GAA)3	9	2010	2018	LSC
p1	(T)9	9	2184	2192	LSC
p3	(GAA)3	9	2822	2830	LSC
p1	(T)8	8	3206	3213	LSC
p3	(ATA)3	9	3546	3554	LSC
p2	(AT)4	8	6373	6380	LSC
p1	(T)8	8	6578	6585	LSC
p1	(A)16	16	7158	7173	LSC
p1	(A)10	10	7485	7494	LSC
p1	(A)9	9	8065	8073	LSC
p1	(T)9	9	8372	8380	LSC
p3	(AAC)3	9	8481	8489	LSC
p1	(A)12	12	8755	8766	LSC
p1	(A)8	8	8899	8906	LSC
p1	(T)14	14	8916	8929	LSC
p1	(T)8	8	9293	9300	LSC
p1	(T)9	9	9645	9653	LSC
p1	(T)8	8	12249	12256	LSC
p1	(T)9	9	13035	13043	LSC
p1	(T)8	8	13060	13067	LSC
p1	(A)8	8	13202	13209	LSC
p4	(AAAT)3	12	13668	13679	LSC
p4	(GGTT)3	12	14256	14267	LSC
p3	(AAC)3	9	14758	14766	LSC
p3	(TTA)3	9	15568	15576	LSC
p2	(CT)4	8	17453	17460	LSC
p3	(AGA)3	9	17658	17666	LSC
p1	(C)8	8	17976	17983	LSC
p1	(T)9	9	18397	18405	LSC
p1	(T)11	11	18503	18513	LSC
p1	(A)8	8	18646	18653	LSC
p2	(AT)4	8	19786	19793	LSC
p2	(AT)5	10	19876	19885	LSC
p3	(TTC)3	9	22130	22138	LSC
p1	(A)8	8	22314	22321	LSC
p1	(T)8	8	26008	26015	LSC
p1	(T)10	10	26112	26121	LSC
p3	(TAT)3	9	27027	27035	LSC
p1	(A)10	10	27263	27272	LSC
p2	(AT)5	10	27350	27359	LSC

p1	(A)8	8	27666	27673	LSC
p3	(TCT)3	9	29018	29026	LSC
p1	(A)8	8	29174	29181	LSC
p4	(AGAT)3	12	29642	29653	LSC
p3	(TTA)3	9	30190	30198	LSC
p1	(A)8	8	30980	30987	LSC
p1	(A)9	9	31074	31082	LSC
p3	(TTA)3	9	31412	31420	LSC
p1	(A)9	9	32076	32084	LSC
p2	(GA)5	10	32307	32316	LSC
p1	(G)8	8	34168	34175	LSC
p2	(GA)4	8	35206	35213	LSC
p1	(A)8	8	36075	36082	LSC
p3	(TCT)3	9	36817	36825	LSC
p3	(TAT)3	9	36868	36876	LSC
p3	(ATG)3	9	38361	38369	LSC
p3	(TTG)3	9	39966	39974	LSC
p3	(GCA)3	9	40259	40267	LSC
p3	(ACC)3	9	41077	41085	LSC
p1	(T)10	10	41737	41746	LSC
p1	(T)8	8	43576	43583	LSC
p1	(A)8	8	44114	44121	LSC
p4	(AACA)3	12	44494	44505	LSC
p3	(TAT)3	9	44511	44519	LSC
p2	(TA)4	8	45489	45496	LSC
p2	(AT)4	8	46144	46151	LSC
p1	(A)8	8	46540	46547	LSC
p1	(A)8	8	46764	46771	LSC
p2	(TA)4	8	47767	47774	LSC
p1	(T)9	9	47861	47869	LSC
p1	(A)8	8	50113	50120	LSC
p1	(T)9	9	51410	51418	LSC
p1	(T)9	9	52355	52363	LSC
p1	(T)10	10	54592	54601	LSC
p3	(AGA)3	9	57720	57728	LSC
p1	(A)8	8	58629	58636	LSC
p1	(A)9	9	61397	61405	LSC
p2	(AC)4	8	61948	61955	LSC
p1	(A)9	9	62590	62598	LSC
p2	(TC)4	8	63271	63278	LSC
p2	(TA)4	8	64042	64049	LSC
p3	(GTA)3	9	64083	64091	LSC
c*	(CCT)3(T)8*	16	65475	65490	LSC
p2	(GT)4	8	66946	66953	LSC

p3	(AAC)3	9	67247	67255	LSC
p3	(ATT)3	9	67522	67530	LSC
p1	(A)9	9	70223	70231	LSC
p1	(T)8	8	73864	73871	LSC
p2	(TA)4	8	77034	77041	LSC
p1	(T)8	8	77326	77333	LSC
p1	(T)8	8	77919	77926	LSC
p1	(A)8	8	78715	78722	LSC
p3	(GGA)3	9	78992	79000	LSC
p1	(T)9	9	79534	79542	LSC
p1	(T)9	9	81327	81335	LSC
p1	(A)8	8	81664	81671	LSC
p2	(CT)4	8	82457	82464	LSC
p2	(TA)4	8	82483	82490	LSC
p3	(TGC)3	9	82699	82707	LSC
p3	(TTA)4	12	82932	82943	LSC
p1	(T)9	9	83203	83211	IRa
p3	(CTT)3	9	83345	83353	IRa
p1	(T)9	9	84785	84793	IRa
p2	(GA)4	8	85431	85438	IRa
p2	(GA)4	8	85443	85450	IRa
p2	(GA)4	8	86430	86437	IRa
p3	(CAA)3	9	86662	86670	IRa
p1	(G)8	8	87194	87201	IRa
p1	(A)9	9	87337	87345	IRa
p2	(GA)4	8	88588	88595	IRa
p3	(TCT)3	9	89277	89285	IRa
p3	(CTT)3	9	89389	89397	IRa
p3	(TGA)3	9	89895	89903	IRa
p1	(A)8	8	89943	89950	IRa
p3	(GAA)3	9	91110	91118	IRa
p2	(TA)4	8	91935	91942	IRa
p3	(AGA)3	9	94510	94518	IRa
p3	(AGA)3	9	95970	95978	IRa
p3	(AAG)3	9	99181	99189	IRa
p1	(C)8	8	99554	99561	IRa
p2	(AG)4	8	101474	101481	IRa
p3	(CTG)3	9	103538	103546	IRa
p2	(CT)4	8	105085	105092	IRa
p3	(CAA)3	9	108572	108580	IRa
p1	(A)8	8	109057	109064	SSC
p1	(A)8	8	109727	109734	SSC
p1	(T)8	8	109747	109754	SSC
p1	(T)10	10	109832	109841	SSC

p3	(AAT)3	9	110173	110181	SSC
p3	(TAA)3	9	110670	110678	SSC
p1	(T)9	9	111256	111264	SSC
p3	(TTA)4	12	111493	111504	SSC
p1	(T)8	8	112710	112717	SSC
p1	(C)8	8	115477	115484	SSC
p1	(T)16	16	117102	117117	SSC
p1	(T)9	9	117906	117914	SSC
p3	(TTC)3	9	118969	118977	SSC
p1	(A)10	10	119490	119499	SSC
p1	(T)8	8	119838	119845	SSC
p3	(AGC)3	9	120099	120107	SSC
p2	(TC)5	10	121364	121373	SSC
p1	(T)8	8	122182	122189	SSC
p2	(AT)4	8	122568	122575	SSC
p1	(T)17	17	123485	123501	SSC
p1	(A)10	10	123674	123683	SSC
p2	(AT)4	8	123942	123949	SSC
p1	(T)9	9	124118	124126	SSC
p3	(CTT)3	9	124633	124641	SSC
p2	(AT)4	8	124749	124756	SSC
p1	(T)9	9	124984	124992	SSC
p1	(T)9	9	125146	125154	SSC
p1	(T)8	8	125263	125270	SSC
p1	(T)9	9	125272	125280	SSC
p3	(TTC)3	9	125757	125765	SSC
p1	(A)9	9	125878	125886	SSC
p1	(A)8	8	125906	125913	SSC
p3	(TCT)3	9	126429	126437	SSC
p1	(T)13	13	126477	126489	SSC
p1	(T)14	14	126654	126667	SSC
p1	(A)10	10	126818	126827	SSC
p3	(TTG)3	9	127371	127379	IRb
p2	(AG)4	8	130859	130866	IRb
p3	(CAG)3	9	132405	132413	IRb
p2	(TC)4	8	134469	134476	IRb
p1	(G)8	8	136390	136397	IRb
p3	(CTT)3	9	136762	136770	IRb
p3	(TTC)3	9	139972	139980	IRb
p3	(TCT)3	9	141433	141441	IRb
p2	(TA)4	8	144009	144016	IRb
p3	(TTC)3	9	144833	144841	IRb
p1	(T)8	8	146001	146008	IRb
p3	(TCA)3	9	146048	146056	IRb

p3	(AAG)3	9	146554	146562	IRb
p3	(AGA)3	9	146666	146674	IRb
p2	(TC)4	8	147356	147363	IRb
p1	(T)9	9	148606	148614	IRb
p1	(C)8	8	148750	148757	IRb
p3	(TTG)3	9	149281	149289	IRb
p2	(CT)4	8	149513	149520	IRb
p2	(TC)4	8	150501	150508	IRb
p2	(TC)4	8	150513	150520	IRb
p1	(A)9	9	151158	151166	IRb
p3	(GAA)3	9	152597	152605	IRb
p1	(A)9	9	152740	152748	IRb

Table S6. Primer sequences at the boundaries between SC and IR regions.

Species	Regions	Forward/Reverse	Primer sequence (5' to 3')
P. rhoeas	LSC-IRa	F	GCTGCTGCCGAATAAACCAA
		R	AAGAGGGTAAATCGGCCACA
	IRa-SSC	F	ACCGAGTTCAACATTAGCCAGA
		R	CGAGCAATTCCGTTGATTGGT
	SSC-IRb	F	TTCAGGGAGGCAGTCTTGTT
		R	ACCGAGTTCAACATTAGCCAG
	IRb-LSC	F	AAGAGGGTAAATCGGCCACA
		R	GAAGCTCCATCCACAAACGG
P. orientale	LSC-IRa	F	GCTGCTGCCGAATAAACCAA
		R	AAGAGGGTAAATCGGCCACA
	IRa-SSC	F	ACCGAGTTCAACATTAGCCAGA
		R	CGAGCAATTCCGTTGATTGGT
	SSC-IRb	F	GCAGGCAGTCTTGTTCAGTC
		R	CCGAGTTCAACATTAGCCAGATT
	IRb-LSC	F	AAGAGGGTAAATCGGCCACA
		R	TGTCGAAGCTCCATCCACAA

Table S7. GenBank accession numbers of dicots with complete chloroplast genome sequences used for phylogenetic analyses.

GenBank acc.	Species	GenBank acc.	Species
NC_029829	*Aconitum chiisanense*	NC_026076	*Paeonia obovata*
NC_033913	*Akebia quinata*	NC_026447	*Panax notoginseng*
NC_029391	*Angelica acutiloba*	NC_029434	*Papaver somniferum*
NC_022457	*Berberis bealei*	NC_008796	*Ranunculus macranthus*
NC_009599	*Buxus microphylla*	NC_027728	*Rheum palmatum*
NC_034950	*Chenopodium album*	NC_029431	*Sabia yunnanensis*
NC_035872	*Circaeaster agrestis*	NC_027224	*Salicornia brachiata*
NC_031446	*Coreanomecon hylomeconoides*	NC_020431	*Salvia miltiorrhiza*
NC_035941	*Decaisnea insignis*	NC_026065	*Sedum takesimense*
NC_029428	*Epimedium sagittatum*	NC_027732	*Sinopodophyllum hexandrum*
NC_029469	*Foeniculum vulgare*	NC_029432	*Stephania japonica*
NC_027669	*Haloxylon persicum*	NC_031849	*Trollius chinensis*
NC_035873	*Kingdonia uniflora*	NC_025339	*Nelumbo nucifera*
NC_029394	*Ligusticum tenuissimum*	NC_032031	*Oxyria sinensis*
NC_008336	*Nandina domestica*	NC_029433	*Pachysandra terminalis*
